# Altered amide proton transfer weighted and diffusion signals in patients with multiple sclerosis: correlation with neurofilament light chain and disease duration

**DOI:** 10.3389/fnins.2023.1137176

**Published:** 2023-04-25

**Authors:** Jing Huang, Yan Liang, Yi Shan, Cheng Zhao, Qiongge Li, Zhiwei Shen, Huiqing Dong, Zhigang Qi, Jie Lu

**Affiliations:** ^1^Department of Radiology and Nuclear Medicine, Xuanwu Hospital, Capital Medical University, Beijing, China; ^2^Beijing Key Laboratory of Magnetic Resonance Imaging and Brain Informatics, Capital Medical University, Beijing, China; ^3^Department of Neurology, Xuanwu Hospital, Capital Medical University, Beijing, China; ^4^Philips Healthcare, Beijing, China

**Keywords:** amide proton transfer, chemical exchange saturation transfer, molecular imaging, multiple sclerosis, neuroinflammation

## Abstract

**Objectives:**

To compare the signal alterations of amide proton transfer (APT), apparent diffusion coefficient (ADC), and fractional anisotropy (FA) in white matter (WM) lesions in multiple sclerosis (MS), compared with healthy controls (HCs), and to investigate the relationships between these changes and clinical measurements such as serum neurofilament light chain (sNfL).

**Materials and methods:**

Twenty-nine patients with relapsing-remitting MS (21 females and 8 males) and 30 HCs (23 females and 7 males) were recruited. APT-weighted (APTw) and diffusion tensor imaging (DTI) data were acquired using a 3.0-T magnetic resonance system. APTw and DTI images were registered to FLAIR-SPIR images and assessed by two neuroradiologists. MTRasym (3.5 ppm), ADC, FA values for MS and HC are calculated using mean values from all regions of interest (ROI). The ROI criteria were: (1) for MS patients, ROI were defined as MS lesions, and each lesion was identified. (2) The WM around each HC’s lateral ventricle (frontal lobe, parietal lobe, and centrum semiovale) was assessed bilaterally. The diagnostic efficacy of MTRasym (3.5 ppm), ADC, and FA in the lesions of MS patients was compared using receiver operating characteristic (ROC) curve analysis. The associations between MTRasym (3.5 ppm), ADC, and FA values and the clinical measurements were investigated further.

**Results:**

The MTRasym (3.5 ppm) and ADC values of brain lesions were increased, while FA values were decreased in patients with MS. The diagnostic area under curve (AUC) of MTRasym (3.5 ppm), ADC, and FA value was 0.891 (95% CI: 0.813, 0.970), 0.761 (95% CI: 0.647, 0.875) and 0.970 (95% CI: 0.924, 1.0), respectively. sNfL was considerably positively correlated with MTRasym (3.5 ppm) (*P* = 0.043, *R* = 0.38) and disease durations were significantly negatively correlated with FA (*P* = 0.046, *R* = −0.37).

**Conclusion:**

Amide proton transfer-weighted (APTw) and DTI are potential imaging methods for assessing brain lesions in patients with MS at the molecular and microscopic levels, respectively. The association between APTw, DTI parameters and clinical factors implies that they may play a role in disease damage monitoring.

## Introduction

Multiple sclerosis (MS) is an inflammatory demyelinating disease of the central nervous system (CNS) that primarily affects young and middle-aged individuals, resulting in a high incidence of neurological impairment and imposing an enormous personal and economic burden on patients, their families, and society (Brownlee et al., 2017; Thompson et al., 2018b). In its early stages, MS has an alternating relapse-remitting clinical course, with relapses typically characterized by acute episodes of neurological impairments, depending on the location of the lesion and the severity of the inflammatory process (Compston and Coles, 2008; Marisa et al., 2021). Although myelin regeneration may repair demyelinating nerve cell lesions in the early stages of MS, self-repair is no longer able to compensate for nerve cell damage and loss as the area of damage expands, resulting in the secondary progressive MS stage. Within 15 to 20 years, 50 to 60 percent of relapsing-remitting MS patients would advance to secondary progressive form if not effectively treated (Noseworthy et al., 2000).

Magnetic resonance imaging (MRI) is an important technique for diagnosing and differentiating MS, as well as monitoring therapeutic outcomes. Utilizing MRI, a variety of morphological information, including focal white matter (WM) lesions, brain volume reduction, gray matter atrophy, and the size and location of MS brain lesions were evaluated (Granziera et al., 2021). However, conventional MRI was unable of quantifying protein weighted information and microstructural cerebral abnormalities, which are essential for the early detection, diagnosis, and treatment of MS (Brownlee et al., 2017).

Amide proton transfer-weighted (APTw) imaging is a kind of chemical exchange saturation transfer (CEST) that detects the exchange rate of protons in amide in free proteins or polypeptide molecules in the body and protons in water to reflect changes in tissue protein content and pH (Zhou et al., 2019). APTw MRI has been used to investigate brain/abdominal tumors, ischemic stroke, and MS (Heo et al., 2019; Joo et al., 2019; Kamimura et al., 2019; Momosaka et al., 2020; Meng et al., 2021; Sartoretti et al., 2021). A recent study using APTw MRI to detect microscopic changes in normally appearing tissue in a mouse model of experimental autoimmune encephalomyelitis (EAE) at early stage suggests that changes in the inflammatory environment may lead to changes in APTw signals, which have the potential to serve as imaging biomarkers (Liu et al., 2019). Dula et al. (2011) found that the APTw signal value of WM lesions were higher than that of healthy brain tissues, and that there was significant variability between individual MS lesions. APTw signals in normal-appearing white matter (NAWM) of spinal cord in MS patients were significantly different from those in healthy controls (HCs), except for WM lesions in the brain, which can provide information regarding biochemical components of spinal cord lesions and disease progression (By et al., 2018). Furthermore, MS lesions and WM hyperintensities of small vessel disease were distinguished by using APTw imaging, but its histogram parameters only provided poor diagnostic performance, implying that more advanced amide proton transfer (APT) signal analysis may be required to help further distinguish MS lesion and WM hyperintensities (Sartoretti et al., 2019). Even though APTw imaging can detect signal changes in MS lesions, it is yet unknown how the APTw signal varies at different clinical phases. Accordingly, an animal study in demyelinating and remyelination stages evaluate the different signal intensities using APTw MRI and indicated that APT signal increases in demyelinating pathological states, but there is no significant change in the state of myelin regeneration (Liu et al., 2019).

Diffusion tensor imaging (DTI) reveals microscopic abnormalities and provides an indirect evaluation of damage to WM fiber tracts. The most significant quantitative parameters are fractional anisotropy (FA) and apparent diffusion coefficient (ADC), which may quantify the diffusion characteristics of WM fiber tracts and the diffusion velocity of water molecules (Krämer et al., 2019), and indirectly reflect the integrity of cell membrane or myelin structure. According to studies, the FA can reflect the microtissue alterations related to axons and has high specificity and sensitivity to changes in WM fiber bundles and the integrity of microtissue (Werring et al., 1999). ADC values of MS lesions were significantly higher than those of healthy subjects (Roychowdhury et al., 2000; Abdoli et al., 2016), the decrease in ADC value may be the result of an inflammatory response, cytotoxicity-induced edema, tumor-related factors whereas an increase in ADC value is mainly caused by vasogenic edema. Decreased FA is more sensitive to axonal damage. However, to our knowledge, no comparative study of APTw and diffusion in MS has been reported.

Neurofilament light chain (NfL) is a cytoskeletal protein of nerve axons that could be detected in cerebrospinal fluid and serum in response to neuronal damage, including neuroinflammation and neurodegeneration (Disanto et al., 2017; Mollenhauer et al., 2020). Serum neurofilament light chain (sNfL) is a promising biomarker of neuroaxonal injury in patients with MS, as it is closely correlated with relapsing activities, disability deterioration, brain shrinkage and therapy response (Barro et al., 2018; Kuhle et al., 2019; Kiani, 2023). However, the relationship between NfL and APTw, DTI parameters remains unclear.

In this study, we investigated and evaluated the efficacy of APTw, ADC and FA in assessing WM lesions in MS patients and correlated the APTw, ADC and FA values with clinical measurements.

## Materials and methods

### Ethical approval and patient consent

This study was approved by the Institutional Review Board of Xuanwu Hospital, Capital Medical University, and written informed consent was obtained from all participants.

### Subjects

We consecutively recruited 29 relapsing-remitting MS (21 females and 8 males) from the Department of Neurology in Xuanwu Hospital, Capital Medical University from January 2020 to September 2021. The relapsing-remitting MS diagnosis was made according to the McDonald criteria (Thompson et al., 2018a). The patients included were relapse-free for at least 3 months and tested for cerebrospinal fluid-oligoclonal IgG bands (CSF-OCB) status. We excluded patients: (1) with history of other neurological diseases; (2) who have treated with disease-modifying medications within 2 weeks before the magnetic resonance (MR) examination. We also enrolled 30 sex- and age-matched healthy controls (HCs; 23 females and 7 males) with no previous history of neurologic dysfunction and with normal findings at neurologic examination and MRI results.

### Image acquisition

A 3.0-T MR system (Ingenia, Philips Healthcare, Best, Netherlands) with an 8-channel head coil was used for the MRI examination. Firstly, an axial FLAIR-SPIR was used to assess the hyperintense brain lesions of MS patients and to exclude HCs with brain lesions. The scanning plane is positioned paralle to a line that connects the corpus callosum’s anterior and posterior parts. Then APTw imaging were performed using a 3D-Dixon turbo spin echo (TSE) imaging sequence with saturation pulse power of 2 μT as well as saturation pulse duration of 2 s. B0 mapping is generated automatically by the DIXON technology with three offset echo time (TE) times in the Philips 3D-APTw sequence, and B0 correction is performed automatically on the scanner. Finally, DTI sequence was performed, and the parameters are shown in Table 1.

**TABLE 1:** Scan parameters of the APTw, 2D T2w TSE, and DTI sequence.

Sequence	Imaging mode	FOV	Thickness (mm)	Gap (mm)	TR/TE (ms)	Matrix	Average	b-values/ Direction	SENSE	Scan during
T2WI-SPAIR-SPIR	2D-TSE	220×201	5	1	8,000/120	308×178	1	–	1.78	2 min 40 s
APTw	3D-Dixon-TSE	230×182	6	0	5,874/7.8	116×91	1	–	1.1	5 min 18 s
DTI-medium-ISO	MS-SE-EPI	256×224	5	1	1,600/2.13	256×224	1	1,000/16	2	8 min 56 s

### Image processing and analysis

APTw images were generated from the host and were registered to FLAIR-SPIR images on the postprocessing workstation of “IntelliSpace Portal” (version 9, Philips Healthcare, Netherlands), which reviewed by two neuroradiologist (YL and JH, with 5 and 11 years of experience in neuroimaging, respectively), and quantitative image analysis was further performed.

In MS group, APTw, ADC and FA maps were fused on FLAIR images using the overlay function in multimodality viewer, and the underlaid FLAIR map was displayed using the alpha blend function to reduce the alpha to 0%. Automatic and manual registration were used to segment the high signal area of MS lesion and generated a volume of interesting (VOI) containing the high signal of the lesion by setting the threshold. To establish consistency of the fused image, several star markers were placed at several typical anatomical locations on each layer of APTw, ADC and FA maps with different thicknesses to assure identical voxels for the same position.

**FIGURE 1:**
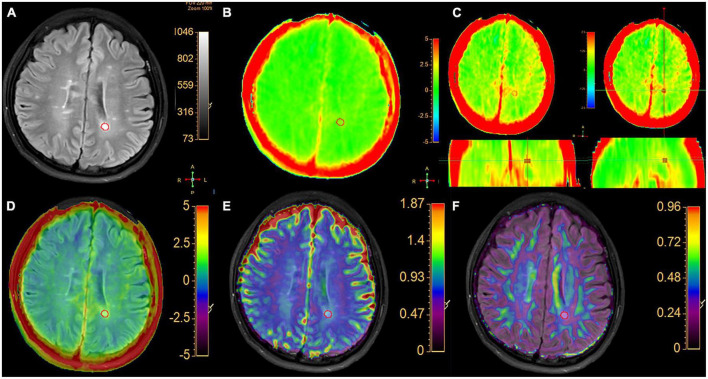
A typical MS patient. **(A)** The FLAIR image and the hyperintense area in white matter were segment semi-automatically delineated regions of interest. **(B)** APTw image with –5 to 5% display range. **(C)** APTw images of three planes with a –2.5 to 2.5% display range. The VOI of lesion is shown in three planes with selected MTRasym (3.5 ppm) values. **(D)** APT and FLAIR image fusion. **(E)** ADC and FLAIR image fusion. **(F)** FA image fused with FLAIR image.

**FIGURE 2:**
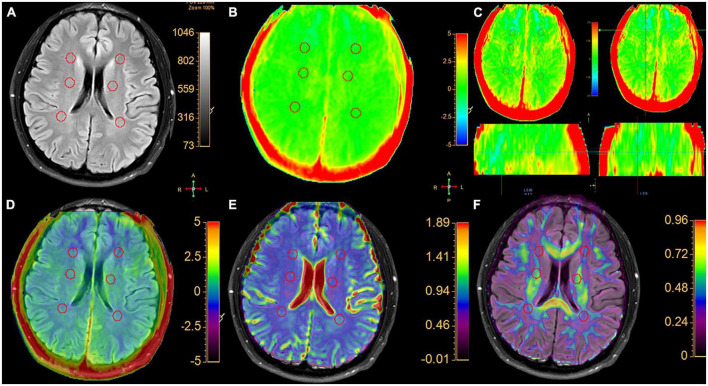
A typical healthy control. **(A)** Six spherical VOIs with a volume of 0.5 cm^3^ were placed bilaterally on normal white matter in the frontal white matter, centrum semiovale and parietal white matter, respectively. **(B)** APTw image with a –5 to 5% display range. **(C)** APTw images of three planes with a –2.5 to 2.5% display range. In three planes, the signal distribution of MTRasym (3.5 ppm) is uniform. **(D)** APT and FLAIR image fusion. **(E)** ADC and FLAIR image fusion. **(F)** FA and FLAIR image fusion.

The VOI requirements were: (1) on axial FLAIR-SPIR images of MS patients, each lesion was identified. The magnetization transfer ratio asymmetry (MTRasym) (3.5 ppm) for MS are calculated using mean values from all lesions (Figure 1). (2) According to the previous reference (Werring et al., 1999), the WM around the lateral ventricle (the frontal lobe, the parietal lobe, and the centrum semiovale) of each HC was selected was assessed bilaterally (Figure 2). MTRasym (3.5 ppm) for HCs are calculated using mean values from six areas. The MTRasym (3.5 ppm) was calculated using the following equation (Abdoli et al., 2016):

MTRasym (3.5 ppm) = {[S_*at*_ (−3.5 ppm)−S_*at*_ (+3.5 ppm)]}/S_0_ (Thompson et al., 2018b).

where S_0_ is the signal intensity without the saturation pulse, S_*sat*_ is the signal intensity after applying the saturation pulse, and MTRasym (3.5 ppm) is the magnetization transfer ratio asymmetry at 3.5 ppm.

The MTRaysm (3.5 ppm), ADC and FA values of the VOI in two groups were recorded in ISP. The VOIs in lesion with a minimum volume greater than 0.1 cm^3^ was selected and the average of MTRaysm (3.5 ppm), ADC and FA was calculated.

### Clinical assessment

The demographic and clinical characteristics, including disease duration, expanded disability status scale (EDSS) score (Kurtzke, 1983), multiple sclerosis severity score (MSSS) (Roxburgh et al., 2005), symbol digit modalities test (SDMT) (Langdon et al., 2012), as well as upper extremity function (9-hole peg test; 9-HPT) and lower limb function (the timed 25-foot walk; T25WT) (Fischer et al., 1999) were recorded by an experienced neurologist (HD, with more than 25 years of experience in neurology) at the time of the MRI.

### Measurement of sNfL

Serum samples were centrifuged at 3,000 r/min for 10 min within 2 h after blood sampling. The samples were immediately frozen and stored at −80°C until they were assayed. The level of sNfL was determined using single molecule array (SIMOA) technology (Quanterix Corporation, Lexington, MA, USA). The quantification limit was 0.315 pg/ml.

### Statistical analysis

The data were analyzed using SPSS (version 17.0, IBM, USA) and R language (version 3.6.1).^1^ The two-sample *t*-test and the Chi-squared test were used to compare the ages and genders of MS and HCs. We used the intraclass correlation coefficient (ICC) to analyze the consistency of MTRaysm (3.5 ppm), FA, and ADC values measured by two neuroradiologists, with the ICC = 0.89, 0.82, and 0.85, respectively, regarded excellent consistency (ICC ≥ 0.75, excellent; 0.60 ≤ ICC < 0.75, good; 0.40 ≤ ICC < 0.60, fair; and ICC < 0.40, poor) (Shieh, 2016). Using receiver operating characteristic (ROC) plot, the area under the curve (AUC) was utilized to evaluate the performance of MTRaysm (3.5 ppm), FA and ADC values in differing the brain lesions of MS patients. Pearson’s correlation analysis was used to analyze the correlations between MTRaysm (3.5 ppm), FA and ADC values and EDSS, MSSS, T25WT, 9-HPT, SDMT scores, disease durations, and sNfL of the MS patients. *P* < 0.05 was considered statistically significant.

### Data availability

The data used to support the findings of this study are available from the corresponding author upon request.

## Results

### Patient’s characteristics

This study included twenty-nine relapsing-remitting MS patients (mean age ± SD: 33.4 years ± 12.0) and thirty HCs (mean age ± SD: 35.2 years ± 11.2). The percentages of males out of all subjects were 27.6% in MS group and 23.3% in HC group, respectively. There were no significant differences in gender and age between MS patients and HCs. Other demographic characteristics of the participants were provided in Table 2.

**TABLE 2:** Demographic and clinical characteristics.

Characteristics	MS (*n* = 29)	HC (*n* = 30)	*P*-values
Mean age (± SD) [years]	33.4 ± 12.0	35.2 ± 11.2	0.542^a^
Sex (M/F)	8/21	7/23	0.708^b^
Median EDSS (range)	2.0 (0–4)	–	–
Median MSSS (± SD)	4.9 ± 2.4	–	–
Median T25FW (± SD)	4.9 ± 1.4	–	–
Median 9HPT (± SD)	25.1 ± 5.2	–	–
Median SDMT (± SD)	44.3 ± 15.8	–	–
Median disease duration (range) (months)	24 (5–132)	–	–
Mean NfL (± SD) (pg/ml)	21.9 ± 8.8	6.1 ± 2.1	< 0.001^a^

^a^Two-sample t-test.

^b^Chi-squared test.

9-HPT, 9-hole peg test; EDSS, expanded disability status scale; HC, healthy control; MS, multiple sclerosis; MSSS, multiple sclerosis severity score; NfL, neurofilament light chain; SDMT, symbol digit modalities test; T25FW, time 25-foot walk. All subjects (HC, MS) were matched for age and sex.

### Data analysis of APTw imaging and DTI

The parameters derived from APT and DTI (mean ± standard deviation) in MS and HC were summarized in Table 3. Two sample *t*-test analysis revealed the APTw values of brain lesions in patients of MS were significantly higher than that in HCs (*P* < 0.001). Additionally, the ADC values of MS patients were significantly higher than those of HCs (*P* < 0.001), the FA values was substantially lower than that HCs (*P* < 0.001).

**TABLE 3:** The parameters derived from APTw and DTI (mean ± standard deviation) in MS and HC.

Parameters	MTRasym (3.5 ppm) (%)	FA	ADC (mm^2^/s)
HC (*n* = 30)	0.64 ± 0.03	0.43 ± 0.03	0.72 ± 0.01
MS (*n* = 29)	0.69 ± 0.02	0.35 ± 0.05	0.74 ± 0.01
*P*-values	< 0.001	< 0.001	< 0.001

ADC, apparent diffusion coefficient; MTRasym (3.5 ppm), asymmetric magnetization transfer rate in 3.5 ppm; FA, fractional anisotropy; HC, healthy control; MS, multiple sclerosis.

### Comparison of the diagnostic performance between APTw imaging and diffusion-weighted imaging (DWI)

**FIGURE 3:**
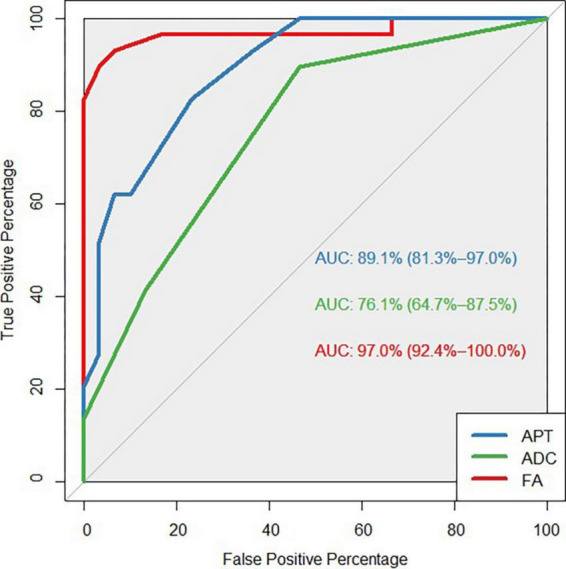
Receiver operating characteristic (ROC) curves showed the ability of APT, ADC, FA values to diagnose MS patients.

The FA value had an AUC of 0.970 (95% CI: 0.924, 1.0), which was higher than the MTRaysm (3.5 ppm) and ADC values. The diagnostic AUCs of MTRaysm (3.5 ppm) and ADC were 0.891 (95% CI: 0.813, 0.970) and 0.761 (95% CI: 0.647, 0.875), respectively (Figure 3 and Table 4).

**TABLE 4:** The parameters of ROC curves.

Variable	AUC	Youden index	95% CI	Cut-off value	Sensitivity (%)	Specificity (%)
ADC	0.761	0.4299	0.647–0.875	> 0.72	89.66	53.33
APT	0.891	0.594	0.813–0.970	> 0.66	82.76	76.67
FA	0.970	0.864	0.924–1.000	≤ 0.37	93.10	93.33

### Correlations between APTw, DTI value and clinical variables

**FIGURE 4:**
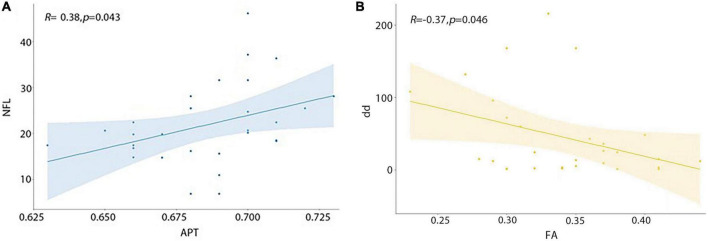
Correlations between APT, FA value and clinical variables. **(A)** MTRasym (3.5 ppm) value was significantly positively correlated with sNfL. **(B)** FA value was significantly negatively correlated with disease duration.

Serum neurofilament light chain (sNfL) was significantly positively correlated with MTRaysm (3.5 ppm) value (*P* = 0.043, *R* = 0.38), while disease durations were significantly negatively correlated with FA value (*P* = 0.046, *R* = −0.37) (Figure 4 and Table 5).

**TABLE 5:** The relationship among MRI indexes and clinical variables (Pearson’s rho/*P*-value).

	MTRasym (3.5 ppm) (%)	FA	ADC (mm^2^/s)
Disease duration (months)	−0.15/0.425	−0.37/0.046	−0.23/0.234
Mean NfL (± SD) (pg/ml)	0.38/0.043	−0.08/0.668	−0.10/0.625

## Discussion

In this study, we evaluated the cellular and molecular abnormalities of brain lesions in MS patients using APTw and DTI sequences. The major findings of this study are as follows: (1) the MTRasym (3.5 ppm) and ADC values of brain lesions in MS patients was significantly higher than those of HCs, while the FA values were significantly lower. (2) The ROC curve revealed that FA and MTRasym (3.5 ppm) had excellent diagnostic performance. (3) MTRasym (3.5 ppm) was found to be significantly positively correlated with sNfL, while the FA value was shown to be significantly negatively correlated with disease duration.

This study included patients with MS who were in the chronic remission stage and had an average disease course of 45 months. The elevated MTRasym (3.5 ppm) of MS lesions in the results is primarily related to axonal damage. To confirm this hypothesis, we tested serum light chain levels of neurofilament protein in patients and age-sex matched healthy volunteers. Regardless of the underlying pathogenic mechanism, the current study reveals that an increase in serum NfL levels is suggestive of continuous neuronal damage, particularly axonal damage. Serum NfL can be used as a biomarker in MS patients to identify subclinical disease activity and to evaluate the effects of drug therapy (Varhaug et al., 2019). A significant positive association was found between MTRasym (3.5 ppm) and NfL, suggesting that the elevated APTw values in MS lesions may be associated with axonal injury, though NfL could not be used as a precise diagnostic tool for MS due to lower specificity. We believe that the combination of NfL and MTRasym (3.5 ppm) could be utilized to objectively determine the degree of neurological damage.

The MTRasym (3.5 ppm) of brain lesions in MS patients is significantly higher than that of normal brain tissue, indicating that APTw imaging could reveal the cytoarchitectural changes that occur in MS lesions during this chronic disease course. Changes in the lactam proton concentration may be responsible for the higher MTRasym (3.5 ppm) observed in lesions of MS patients. Protein accumulation and concentration in activated microglia surrounding chronic active MS lesions, or increased protein degradation during axonal injury, followed by a high concentration of active peptide, may be responsible for the increased MTRasym (3.5 ppm) (Absinta et al., 2019; Elliott et al., 2019).

The findings indicated that MTRasym (3.5 ppm), ADC and FA had significant diagnostic value, with FA having the highest AUC of 0.97 in differentiating MS lesions from normal brain tissue in this study, which is consistent with the previous research (Klistorner et al., 2018). Without considering the complexity of biological tissue microstructure, such as the cell membrane and cell density, ADC values reflect varying degrees of influence on water molecule diffusion, resulting in water molecule movement deviating from the Gaussian distribution, so ADC values in diagnosis of efficiency are lowest in this study.

There are several limitations in our study. First, this is a cross-sectional study with a small sample size; a larger sample size longitudinal study is required to validate the current conclusion. Second, due to the technical limitations of the single-layer acquisition protocol, the APTw images in this study only obtained seven-layer imaging, making it impossible to investigate the MTRasym (3.5 ppm) alterations of lesions in all brain regions, and the small APTw effect resulted in low spatial resolution. In the next study, more advanced APTw imaging acquisition or analysis methods should be used to measure APT values more comprehensively and precisely. Finally, this study evaluated the difference in APTw values between relapsing-remitting MS patients and HCs, with no other demyelinating diseases patients included. In future work, patients with other demyelinating diseases, for example, neuromyelitis optica spectrum disorders (NMOSD) should be included to determine whether the APTw value has the potential ability to distinguish MS and NMOSD.

## Conclusion

The current study evaluated the cellular and molecular imaging of APTw and DTI for the brain lesions in patients with MS. Our preliminary results indicate that these two MRI methods provide additional quantitative diagnostic information for clinical use. APTw and DTI could help us understand the pathological changes in MS with more sensitively and specificity, and the high correlation between MTRasym (3.5 ppm), FA and clinical factors suggests that APT may play a role in monitoring disease impairment.

## Data availability statement

The original contributions presented in this study are included in the article/supplementary material, further inquiries can be directed to the corresponding author.

## Ethics statement

The studies involving human participants were reviewed and approved by the Institutional Review Board of Xuanwu Hospital, Capital Medical University, and written informed consent was obtained from all participants. The patients/participants provided their written informed consent to participate in this study.

## Author contributions

JL and JH designed experiments. JH, YL, and HD performed the experiments. ZS provided technical advice. QL, ZQ, and CZ assisted with collecting data. JH and ZS wrote the manuscript. All authors contributed to the article and approved the submitted version.

## Footnotes

1
https://www.r-project.org/
